# Dielectric Nanoparticle Dimers on a Mirror for Enhanced Photoluminescence of Quantum Dots

**DOI:** 10.1002/nap2.70319

**Published:** 2026-10-14

**Authors:** Rafael Ramos Uña, Braulio García‐Cámara, Ángela I. Barreda

**Affiliations:** ^1^ Department of Electronic Engineering University Carlos III of Madrid Avda. de la Universidad Leganés Spain

**Keywords:** dielectric nanoparticles, image theory, metallic nanoparticles, nanoparticle‐on‐a‐mirror, quantum emitters

## Abstract

Nanoantennas for enhancing quantum emitters' response have attracted considerable attention, particularly those based on hybrid metal‐dielectric configurations. However, it remains a challenge to simultaneously enhance excitation and emission while maintaining directionality. In this work, novel hybrid metal‐dielectric designs based on the nanoparticle‐on‐mirror architecture are analyzed to maximize the photoluminescence of quantum dots. The proposed nanostructure consists of a silicon nanocylinder dimer placed on an aluminum substrate separated by a thin spacer layer embedding randomly distributed CdSe/ZnS quantum dots. By combining a semianalytical approach based on image theory with numerical simulations, it is demonstrated that the interaction between the nanoparticles in the dimer, which gives rise to new modes, can lead to enhanced photoluminescence. The analysis is further extended to periodic metasurfaces composed of arrays of these dimer unit cells, showing that the enhanced excitation and directional emission are preserved in large‐area devices. These results are relevant for the development of single‐photon sources, single‐molecule detection, and high‐brightness nanophotonic light sources for displays and bioimaging technologies.

## Graphical abstract

Hybrid metal–dielectric nanoantennas based on nanoparticle‐on‐mirror architectures are proposed to enhance quantum dot photoluminescence. Silicon nanocylinder dimers on aluminum substrates enable simultaneous excitation enhancement and directional emission through strong light–matter interactions. The results of this work offer promising applications in single‐photon sources, sensing, displays, and bioimaging technologies.

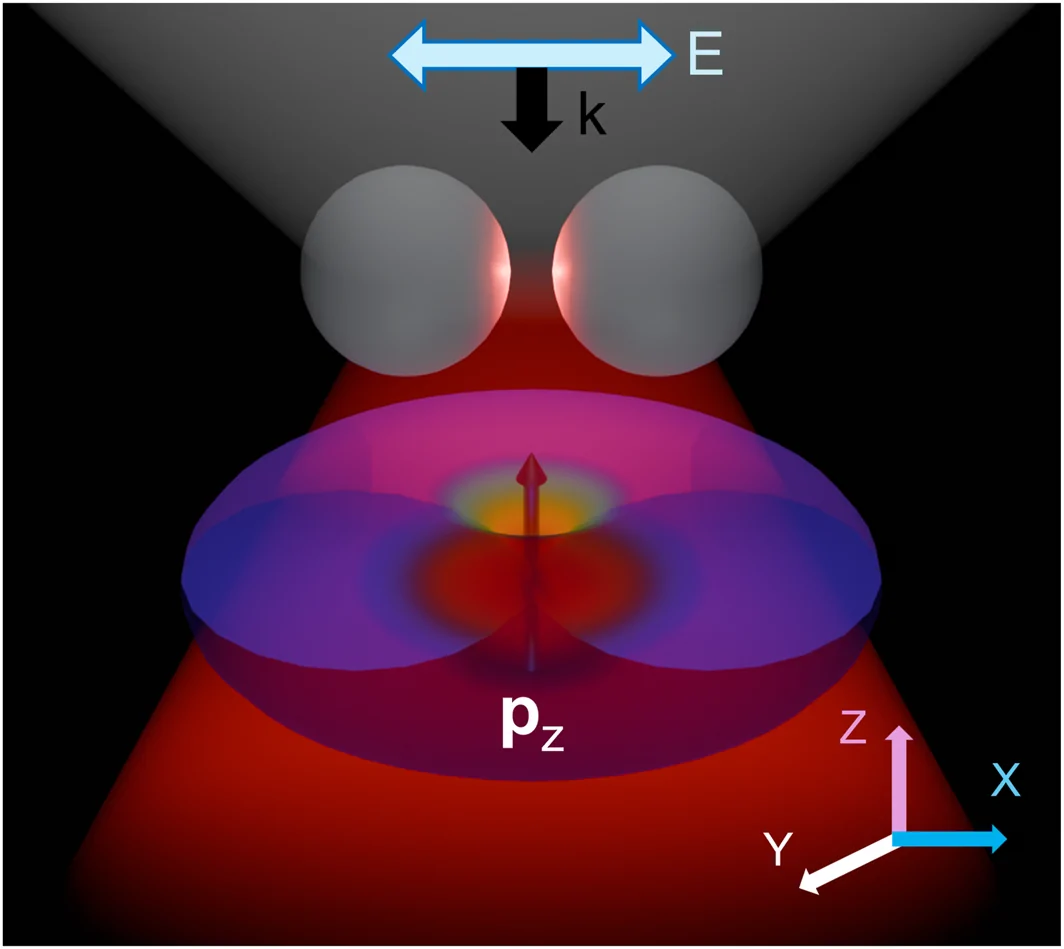

## 1 Introduction

Controlling and enhancing light emission of quantum emitters remains a key challenge in the development of efficient single‐photon sources [1, 2, 3]. To address this challenge, various strategies have been explored, including coupling quantum emitters to photonic nanostructures to enhance their photoluminescence (PL) [4, 5].

In particular, nanoantennas have proven to be powerful tools for manipulating light–matter interactions at the nanoscale, enabling improved radiative coupling and directional emission control. Nanoantennas can be composed of either metallic or dielectric materials, each offering specific advantages and limitations [6, 7]. Metallic nanostructures provide strong electromagnetic field confinement but suffer from absorption losses [8, 9], whereas dielectric nanoantennas exhibit low ohmic losses and allow improved control over emission directivity through the interplay of electric and magnetic modes [10, 11], albeit with weaker field confinement than their plasmonic counterparts. More recently, hybrid nanoantennas have emerged as a promising alternative by combining the advantages of both material platforms [6, 7, 12, 13, 14, 15, 16, 17, 18].

However, designing nanostructures that simultaneously maximize excitation and emission efficiencies, as well as emission directivity, while remaining compatible with scalable technological platforms, remains an open challenge.

Nanoparticle‐on‐a‐mirror (NPoM) configurations, in which emitters are placed within the spacer formed between a nanoparticle (NP), either metallic or dielectric, and a metallic substrate [19, 20, 21, 22, 23, 24, 25], have demonstrated remarkable performance. In these systems, the coupling between NP resonances and their mirror image enhances PL by strengthening light–matter interactions while also enabling directional emission.

State‐of‐the‐art NPoM platforms using plasmonic NPs have shown significant improvements across different studies, including magnetic Purcell enhancements of up to 76 [20], tenfold increases in collection efficiency [19], and field confinement factors approaching $10^{6}$ [22]. However, the strong nonradiative losses inherent to plasmonic structures limit their overall efficiency.

In contrast, dielectric NPs on metallic mirrors can provide fluorescence enhancement factors as high as 654, with average enhancements of 157.4 in emission, 7.3 in excitation, and 1.31 in directionality [25].

Enhancing the excitation rate of quantum emitters requires strong electromagnetic field confinement at their location, which can be achieved through resonant modes capable of generating intense hot spots. Strong electromagnetic field confinement is also required to enhance emission. In addition, efficient collection of the emitted radiation by the optical setup benefits from good directionality.

In this context, interactions between the modes supported by the NPs in a dielectric dimer give rise to new modes [26, 27, 28], which can enhance the PL of quantum emitters. However, dielectric NP dimers on metallic substrates remain largely unexplored.

Here, we investigate the PL of quantum dots randomly distributed within the spacer between a dimer of high refractive index dielectric NPs and a metallic mirror. By varying the interparticle distance, we reveal how the gap controls excitation and emission processes, as well as the resulting emission directionality. To gain physical insight, we combine image theory and multipolar decomposition to identify the modes governing the system response.

We further extend this concept to periodic metasurfaces composed of these unit cells, bridging the gap between individual nanoantennas and large‐area devices. Our results establish dielectric dimers on metallic substrates as an effective strategy to enhance light–matter interactions while preserving directionality, paving the way for advanced quantum light sources, fluorescence‐based sensing, and integrated nanophotonic technologies.

## 2 Nanostructure Design

The proposed nanoantenna consists of a silicon nanocylinder dimer, with radius R=95 nm and height H=50 nm, placed on a metallic aluminum substrate and separated from it by a $SiO_{2}$ spacer layer of 10 nm, as shown in Figure 1. The optical constants of all materials were taken from the Palik handbook [29] (see Supporting Information S1: Section S1 [SI‐S1]). The silica spacer layer embeds CdSe/ZnS quantum dots (QDs), which present strong optical absorption around 570 nm and an emission peak centered at 650 nm [25].

**FIGURE 1:**
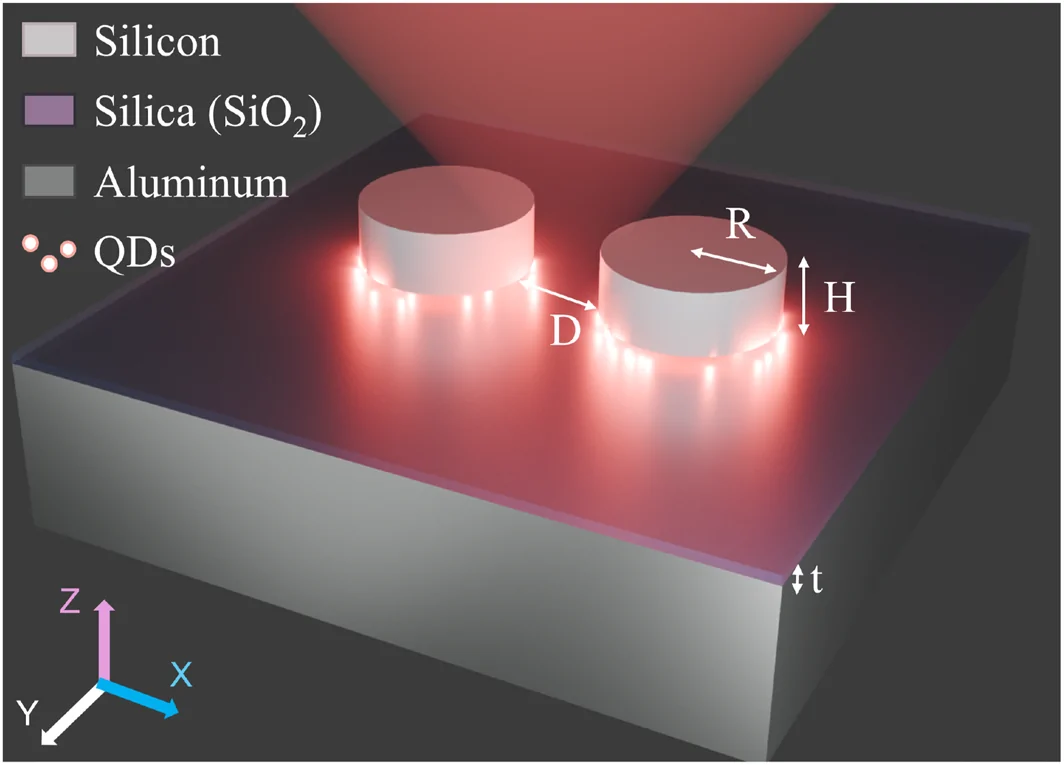
Schematic representation of the nanoantenna configuration consisting of two silicon nanocylinders with radius R and height H, separated by a distance (gap) D and positioned on an aluminum substrate. A silica spacer layer of thickness t is placed between the nanocylinders and the substrate, hosting the QDs.

This geometry was selected because, while spherical NPs can support interaction‐induced novel modes, their minimal contact area with the substrate limits field confinement in the spacer region. In contrast, cylindrical NPs provide a larger contact interface, which promotes stronger hot‐spot formation within the QD region, leading to enhanced field confinement and improved light–matter interaction. In addition, nanocylinders are well suited for top‐down fabrication, because their dimensions, positions, and interparticle separations can be lithographically defined. Compared with more complex geometries such as nanorings [25], solid nanocylinders require the lithographic definition of only a single outer boundary. In contrast, nanorings require accurate definition of both the inner and outer boundaries, together with precise control of the resulting ring width and their relative alignment. Therefore, solid nanocylinders reduce the number of critical geometrical parameters that must be accurately controlled during fabrication. Nevertheless, the realization of very small and uniform lateral gaps remains challenging. Therefore, the minimum interparticle separation considered in this work was restricted to D=10nm, which represents an experimentally accessible value for lithographically defined silicon nanostructures [30, 31]. For completeness, a detailed study of the new modes arising in a dimer of spherical silicon NPs due to their mutual interaction is provided in the Supporting Information S1: SI‐S2 [26].

Building upon the previously presented design, metasurfaces with unit cells composed of dimers of silicon NPs have also been studied. Periodic arrays of silicon nanoresonators are also compatible with established lithographic fabrication techniques [32].

## 3 Numerical Analysis

To analyze the PL enhancement of the proposed structures, both analytical and numerical methods have been used. In particular, Green's function formalism, together with image theory and multipolar decomposition, has been used to obtain the semianalytical results presented in this work, providing insights into the modes responsible for the observed PL enhancement. In addition, numerical simulations based on the finite‐difference time‐domain (FDTD) method were carried out using the commercial software Lumerical.

### 3.1 Excitation Enhancement

The excitation enhancement was evaluated according to the principle that the excitation rate is proportional to the local electric‐field intensity at the emitter location [33]. It can therefore be expressed as

(1) $$\eta_{\text{exc}}=\frac{{\left|E\right|}^{2}}{{\left|E_{0}\right|}^{2}}$$

where E is the electric field at the emitter's position and $E_{0}$ is the electric field at the same location in the absence of the nanoantenna. Further details on the simulation setup to obtain this parameter are given in the Supporting Information S1: SI‐S3.

### 3.2 Emission Enhancement and Directionality

To account for the random spatial distribution and arbitrary dipole orientations ($p_{x}$, $p_{y}$, and $p_{z}$) of the QDs within the spacer layer, the Lorentz reciprocity principle was used [25]. According to this principle, the emission of a dipole located at a given position into a specific far‐field channel can be determined from the reciprocal electric field generated at the dipole position by illumination from the corresponding far‐field direction [34]. This approach enables the emission response of the QD ensemble to be evaluated while accounting for both the spatial distribution and the different dipole orientations.

In the simulations, the nanoantennas were excited using plane‐wave illumination at normal incidence with two orthogonal linear polarizations. The resulting near‐field distribution was sampled on a plane located at the center of the spacer using an array of point monitors. These monitors were uniformly distributed over a square area with side length equal to the nanoantenna diameter, as detailed in the Supporting Information S1: SI‐S4.

Within this reciprocity framework, the power obtained at each monitor from the projection of the electric field along a given axis i is equivalent to that radiated by an electric dipole oriented along axis i and located at the monitor position. The emission enhancement is then obtained by integrating the collected power over all dipole orientations (i=x,y,z) and across the spatial distribution of monitors, averaging over the two excitation polarizations, and normalizing to the reference case without the nanoantenna [34]. Because the nanostructure is illuminated at normal incidence, the emission is directed upwards (i.e., out‐of‐plane).

Finally, to analyze the emission directionality, we used the reciprocity principle using the same simulation setup as described earlier [25]. The directionality is defined as the fraction of light collected in the upward region within a given numerical aperture of the collection system $\left({NA}_{\text{col}}\right)$ and is numerically evaluated as

(2) $$D_{\text{em}}=\frac{\int_{0}^{2\pi }\int_{0}^{\theta_{\text{col}}}p\left(\theta ,\phi \right)\sin\;\theta \;d\theta \;d\phi }{\int_{0}^{2\pi }\int_{0}^{\pi }p\left(\theta ,\phi \right)\sin\;\theta \;d\theta \;d\phi }$$

where p(θ,ϕ) denotes the angular power radiated into a given solid angle, parametrized by the angles θ and ϕ, and $\theta_{\text{col}}$ is defined as $\theta_{\text{col}}=\sin^{-1}\left({NA}_{\text{col}}\right)$. For the directivity calculation, only emission along a single direction is considered, namely, the upward direction (θ=0). In this limit, the collection angle approaches zero $\left(\theta_{\text{col}}\to 0\right)$, and the directivity reduces to the evaluation of the angular power in the normal direction rather than an angular integration.

## 4 Results and Discussion

### 4.1 Nanoantenna Dimer for PL Enhancement

One of the main challenges in PL enhancement is achieving efficient excitation and emission of the emitters simultaneously. For single nanocylinders, it is not straightforward to obtain resonances at both the excitation and emission wavelengths, especially when the spectral separation is small, as in the case of CdSe/ZnS QDs. To address this issue, we propose considering NP dimers instead of isolated structures. Because of the interaction between the two components of the dimer, additional modes can emerge, enabling resonances at both excitation and emission wavelengths through appropriate design of the interparticle gap and NP size.

#### 4.1.1 Scattering Efficiency

Figure 2 shows the scattering efficiency $Q_{\mathrm{sca}}$, calculated as described in the Supporting Information S1: SI‐S5, for a single nanocylinder (Figure 2a) and for NP dimers with different interparticle gap distances. In particular, small gaps (D=10 nm) and large gaps (D=100 nm) are presented in Figure 2b,c, respectively. To identify the modes responsible for the resonances observed in the spectra, the multipolar decomposition was performed.

**FIGURE 2:**
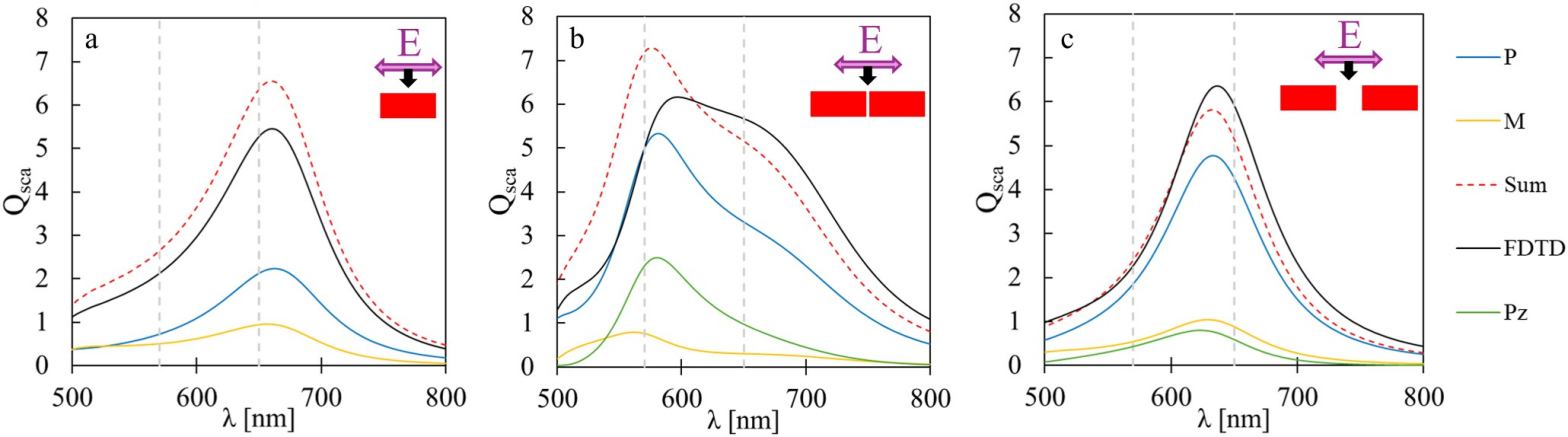
Multipolar decomposition of the nanoantenna for (a) an isolated NP and for dimers with (b) D=10 nm and (c) D=100 nm. The vertical dashed lines indicate the excitation (570 nm) and emission (650 nm) wavelengths. The black solid line corresponds to the scattering efficiency obtained from numerical simulations (FDTD, Lumerical), whereas the red dashed line represents the calculated efficiency from the electric and magnetic dipolar contributions. The individual contributions from the electric and magnetic dipoles are shown by the blue and yellow solid lines, respectively. For the dimers, the green line highlights the contribution of the out‐of‐plane electric dipole $\left(p_{z}\right)$ to the scattering efficiency. The structures were illuminated by a plane wave propagating along the negative direction of the z‐axis and linearly polarized along the x‐axis, as shown in the insets.

Analytically reproducing the scattering efficiency from the excited multipoles in NP dimers on a metallic substrate is not straightforward. To address this, we used the image theory approach, in which the metallic substrate is replaced by the mirror images of the NPs.

First, the system, consisting of either a single dielectric NP or a dimer separated from the metallic substrate by a dielectric spacer, was illuminated by a plane wave linearly polarized along the x‐axis (p‐polarization) and propagating along the negative z‐direction.

The dipolar moments of each NP were obtained from the displacement current density J(r) inside the particle [35].

The electric dipole moment is given by

(3) $$p=\frac{i}{\omega }\int J\left(r\right)\;j_{0}\left(kr\right)\;dV,$$

The magnetic dipole moment is given by

(4) $$m=\frac{3}{2}\int \left[r\times J\left(r\right)\right]\frac{j_{1}\left(kr\right)}{kr}\;dV,$$

The toroidal dipole moment is given by

(5) $$t=\frac{ik^{2}}{2\omega }\int \left[3\;r\left(r\cdot J\left(r\right)\right)-r^{2}J\left(r\right)\right]\frac{j_{2}\left(kr\right)}{kr}\;dV,$$

where $j_{n}\left(kr\right)$ are the spherical Bessel functions of order n, k is the wavenumber, and ω is the angular frequency. In the analysis, the toroidal dipole contribution is included within the effective electric dipole moment.

Once the dipolar moments were obtained, the scattered field from the NPs on the substrate was calculated using image theory. Each NP was first approximated by equivalent electric and magnetic dipoles located at its center, derived from the calculated multipolar moments, as shown in Figure 3a,c for a single NP and a dimer, respectively. It is important to note that, under the illumination conditions considered in this work, only the $p_{x}$ and $m_{y}$ moments are excited in the case of an isolated NP. In contrast, for the dimer configuration, an additional out‐of‐plane electric dipole moment $p_{z}$ emerges because of the interaction between the NPs, as discussed later.

**FIGURE 3:**
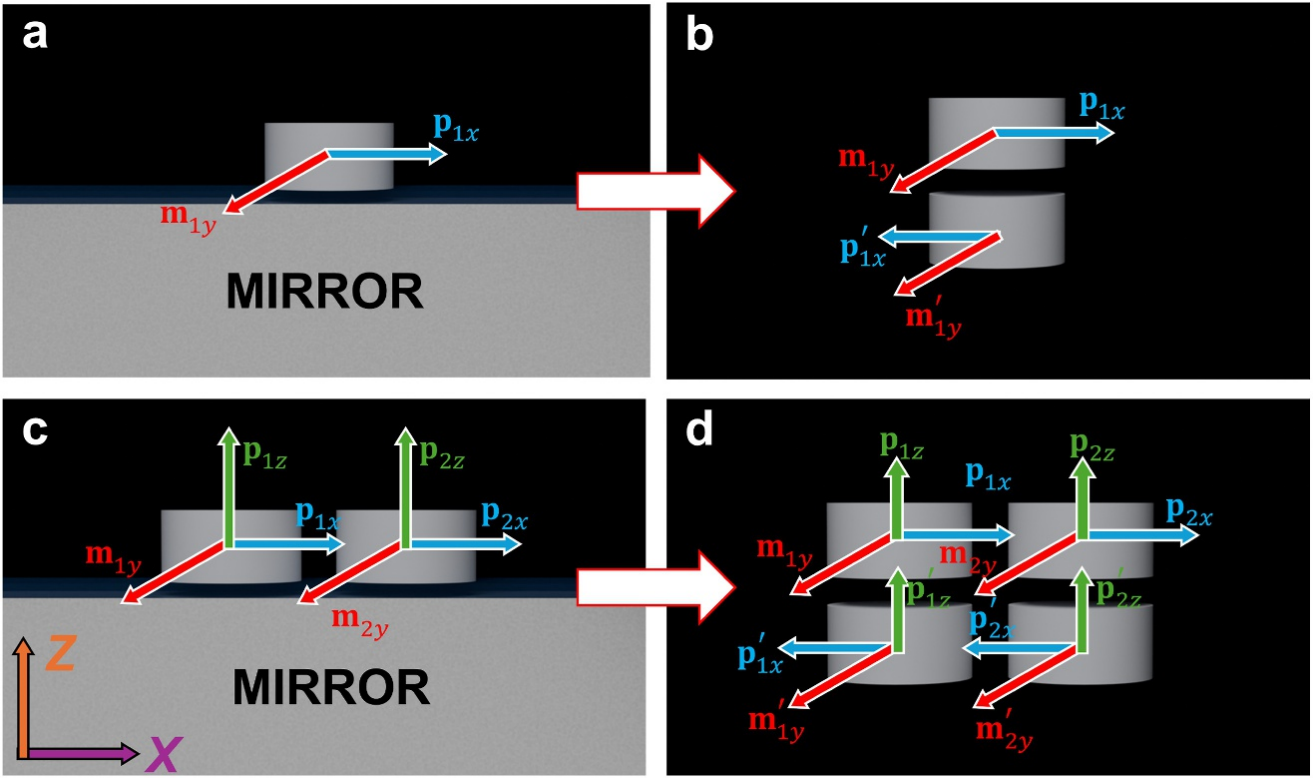
(a) Dielectric NP on a metallic substrate. (b) Equivalent system of (a) obtained using image theory. (c) Dielectric NP dimer on a metallic substrate. (d) Equivalent system of (c) based on image theory. The induced electric (p) and magnetic (m) dipole moments of both the real and image NPs are shown, with the image dipole moments denoted by $p^{\prime }$ and $m^{\prime }$.

Subsequently, the substrate was replaced by the image dipoles ($p^{\prime }$, $m^{\prime }$) corresponding to the real dipoles (p, m), following the relations given in the following equations and illustrated in Figure 3b,d for the single NP and the dimer, respectively:

(6) $$\begin{array}{cccccc} p_{1x}^{\prime } & =-p_{1x}, & \;p_{1y}^{\prime } & =-p_{1y}, & \;p_{1z}^{\prime } & =p_{1z},\\ m_{1x}^{\prime } & =m_{1x}, & \;m_{1y}^{\prime } & =m_{1y}, & \;m_{1z}^{\prime } & =-m_{1z}. \end{array}$$

Notably, the distance between the NP and its mirror image accounted for the spacer thickness. Consequently, the separation between the real and image dipoles was given by the NP diameter plus twice the spacer thickness, that is, 2R+2t. However, the effect of the spacer refractive index on the electromagnetic response was neglected in a first‐order approximation because of its small thickness.

Once all dipolar contributions were defined, the total electric field was obtained by superposition of the fields radiated by each dipole. In the dipolar approximation, the electric field at an observation point r can be written as

(7) $$E\left(r\right)=\sum_{\alpha }\left[E_{p}^{\alpha }\left(r\right)+E_{m}^{\alpha }\left(r\right)\right],$$

where $E_{p}^{\alpha }\left(r\right)$ and $E_{m}^{\alpha }\left(r\right)$ denote the electric fields generated by the electric and magnetic dipoles associated with NP α, respectively. Their far‐field expressions are given by Equations (8) and (9). In the mirror‐image approach, the summation index α runs over both the real dipoles and their corresponding image dipoles, thereby accounting for the substrate contribution through additional dipolar sources located at the mirrored positions:

(8) $$E_{p}^{\text{ff}}\left(r\right)\approx \frac{k^{2}e^{ik|r-r_{d}|}}{4\pi \varepsilon_{h}\varepsilon_{0}|r-r_{d}|}\left[p\left(r_{d}\right)-\left(u_{r}\cdot p\left(r_{d}\right)\right)u_{r}\right]$$

(9) $$E_{m}^{\text{ff}}\left(r\right)\approx -Z\frac{k^{2}e^{ik|r-r_{d}|}}{4\pi |r-r_{d}|}\left[u_{r}\times m\left(r_{d}\right)\right].$$

$r_{d}$ denotes the dipole location and $u_{r}=\frac{r-r_{d}}{\left|r-r_{d}\right|}$ is the unit vector pointing from the dipole to the observation point. The quantity $Z={\left(\mu_{0}\mu_{h}/\varepsilon_{0}\varepsilon_{h}\right)}^{1/2}$ represents the impedance of the surrounding medium. Here, $\varepsilon_{0}$ and $\mu_{0}$ denote the dielectric permittivity and magnetic permeability of vacuum, respectively, whereas $\varepsilon_{h}$ and $\mu_{h}$ are the relative dielectric permittivity and magnetic permeability of the embedding medium.

Finally, the scattering cross‐section was obtained by integrating the electric‐field intensity over the upper hemisphere, that is, over the angular range 0≤θ≤π/2 and 0≤ϕ≤2π, corresponding to radiation emitted into the half‐space above the substrate. The scattering efficiency was then calculated by normalizing the scattering cross‐section to the geometrical cross‐section of the NP. For dimers, the normalization was performed using twice the geometrical cross‐section of a single NP. Therefore, the electric and magnetic dipolar contributions shown in Figure 2 represent the scattering arising from the dipolar fields of the individual nanoparticle in Figure 2a and from the coherent superposition of the dipolar fields of the two individual disks in Figure 2b,c while accounting for the effect of the metallic substrate through their respective image dipoles.

As shown in Figure 2a, the single NP exhibits a resonance close to the emission wavelength (650 nm), where the scattering is primarily governed by the electric dipole contribution, with a smaller contribution from the magnetic dipole. In contrast, the multipolar decomposition of the dimers (Figure 2b,c) shows a more complex behavior. In the case of the smaller gap dimer (D=10 nm), a broad electric dipole resonance spans both the excitation and emission wavelengths. By comparing the scattering efficiency associated with the total electric dipole (including the x, y, and z components) and that corresponding solely to the z component (blue and green curves, respectively), it is evident that the $p_{z}$ contribution is significant at these wavelengths, mainly at the excitation wavelength. This behavior arises from the strong interaction between the NPs, as discussed in the Supporting Information S1: SI‐S2.

For the larger gap dimer (D=100 nm), the multipolar decomposition closely resembles that of an isolated NP. At the emission wavelength, a resonance is observed, primarily dominated by the electric dipole, although a non‐negligible magnetic contribution is also present. The persistence of a $p_{z}$ component indicates that interparticle coupling remains, albeit weaker than in the small‐gap case.

These results highlight the crucial role of the dimer configuration in enabling resonances at both the excitation and emission wavelengths. Furthermore, in the NPoM configuration, the modes supported in the spacer exhibit a strong out‐of‐plane electric‐field component $\left(E_{z}\right)$, leading to significant field enhancement within the gap. The interparticle coupling in the dimer induces additional out‐of‐plane electric dipole moments, which modify the modal distribution and strengthen the coupling to gap‐confined modes, thereby further enhancing the electromagnetic field concentration within the spacer.

From these results, it can be observed that the combination of the mirror‐image approach and multipolar decomposition provides clear insights into the modes responsible for the resonances in the scattering efficiency spectrum. The agreement between the numerical and semianalytical results is remarkable, particularly for the isolated NP and for large interparticle gaps. For small gaps between the silicon NPs, the mismatch can be attributed to the possible contribution of higher‐order multipolar terms.

### 4.2 Near‐Field

The near‐field distribution was evaluated for an isolated NP and for both dimer configurations: D=10 nm and D=100 nm. In this sense, Figure 4 shows the normalized electric‐field amplitude $\left|E|/|E_{0}\right|$ at the excitation wavelength (left column) and at the emission wavelength (right column). The near‐field maps were calculated in the central XZ plane bisecting the nanostructures.

**FIGURE 4:**
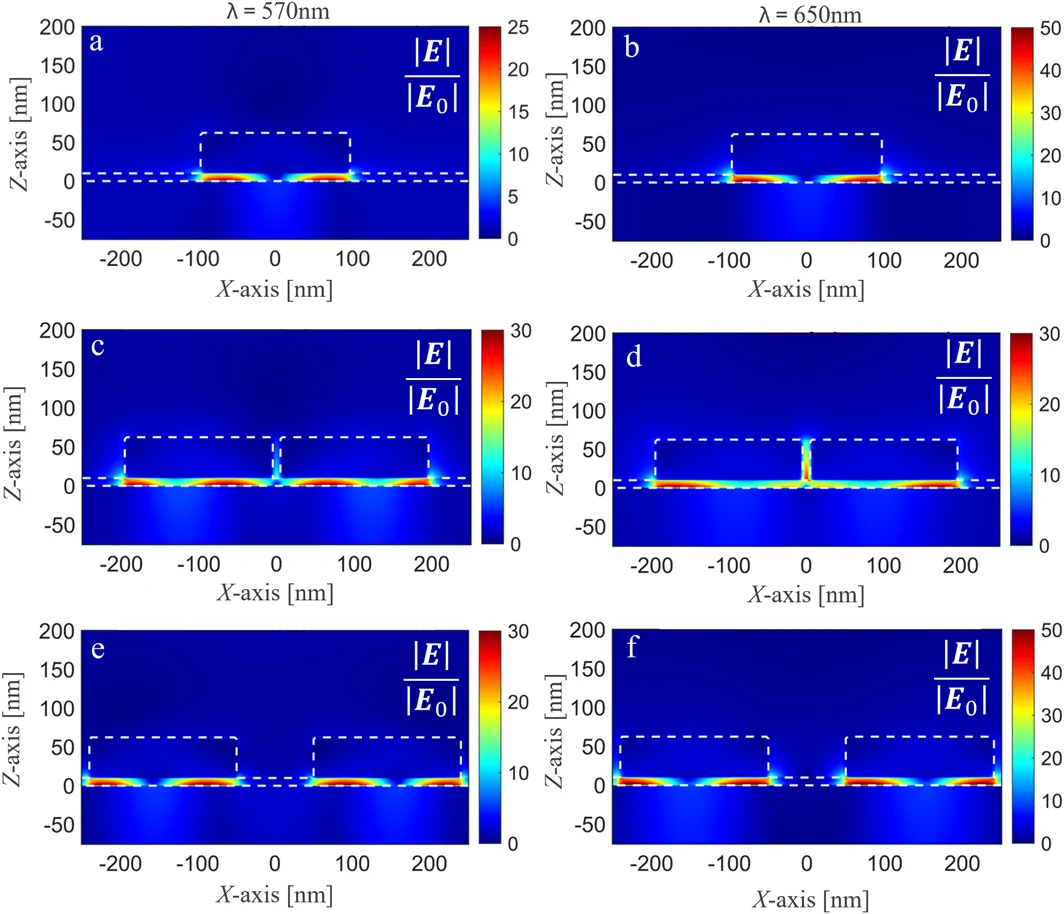
Near‐field electric‐field enhancement, $\left|E|/|E_{0}\right|$, in the XZ plane bisecting the nanostructures, for an isolated NP (a, b) and for dimer configurations with interparticle gaps D=10 nm (c, d) and D=100 nm (e, f). The excitation wavelength (570 nm) is shown in panels (a), (c), and (e), whereas the emission wavelength (650 nm) is shown in panels (b), (d), and (f). The structures were illuminated by a plane wave linearly polarized along the x‐axis and propagating along the −z‐direction. White dashed lines indicate the NP positions and the spacer region. Color scales differ between panels for clarity.

At λ=570 nm (Figure 4a,c,e), the hot spots are strongly confined within the silica spacer layer. For the dimers, the electric field is enhanced compared with the isolated NP due to the coupling between the NPs, which gives rise to additional modes associated with z‐oriented dipole moments. This effect becomes more pronounced as the gap size decreases. Although the maximum electric‐field intensity is similar for both D=10 nm and D=100 nm, their field distributions differ. For D=10 nm, hot spots are distributed throughout the entire spacer region beneath the NPs, whereas for D=100 nm, regions with little or no electric‐field enhancement are observed within the spacer layer. This behavior is particularly evident beneath the center of the NPs, where a dark blue region indicates almost no field enhancement.

On the other hand, at λ=650 nm (Figure 4b,d,f), the maximum electric‐field enhancement is observed for the isolated NP and the dimer with a gap of D=100 nm. In addition to the differences in the maximum enhancement, the electric‐field distribution changes. For the isolated NP and the dimer with D=100 nm, the hot spots remain strongly localized within the spacer layer. In contrast, the dimer with D=10 nm exhibits a more delocalized field distribution, indicating that the reduced interparticle distance enhances the electric field not only within the spacer layer but also in the gap region between the NPs.

### 4.3 Excitation Enhancement

The average value of the excitation enhancement (Equation 1) was evaluated for QDs positioned beneath the NPs, $\langle \eta_{\text{exc}}\rangle$. It was assumed that the QDs are distributed within a square region of the spacer layer with side length equal to the disk diameter. In the case of dimers, the QDs are located within two such regions, one centered around each NP. Further details regarding the simulation methodology are provided in the Supporting Information S1: SI‐S3.

The average excitation enhancement results for both a single NP and the proposed dimer configurations, considering different gap sizes, are presented in Figure 5.

**FIGURE 5:**
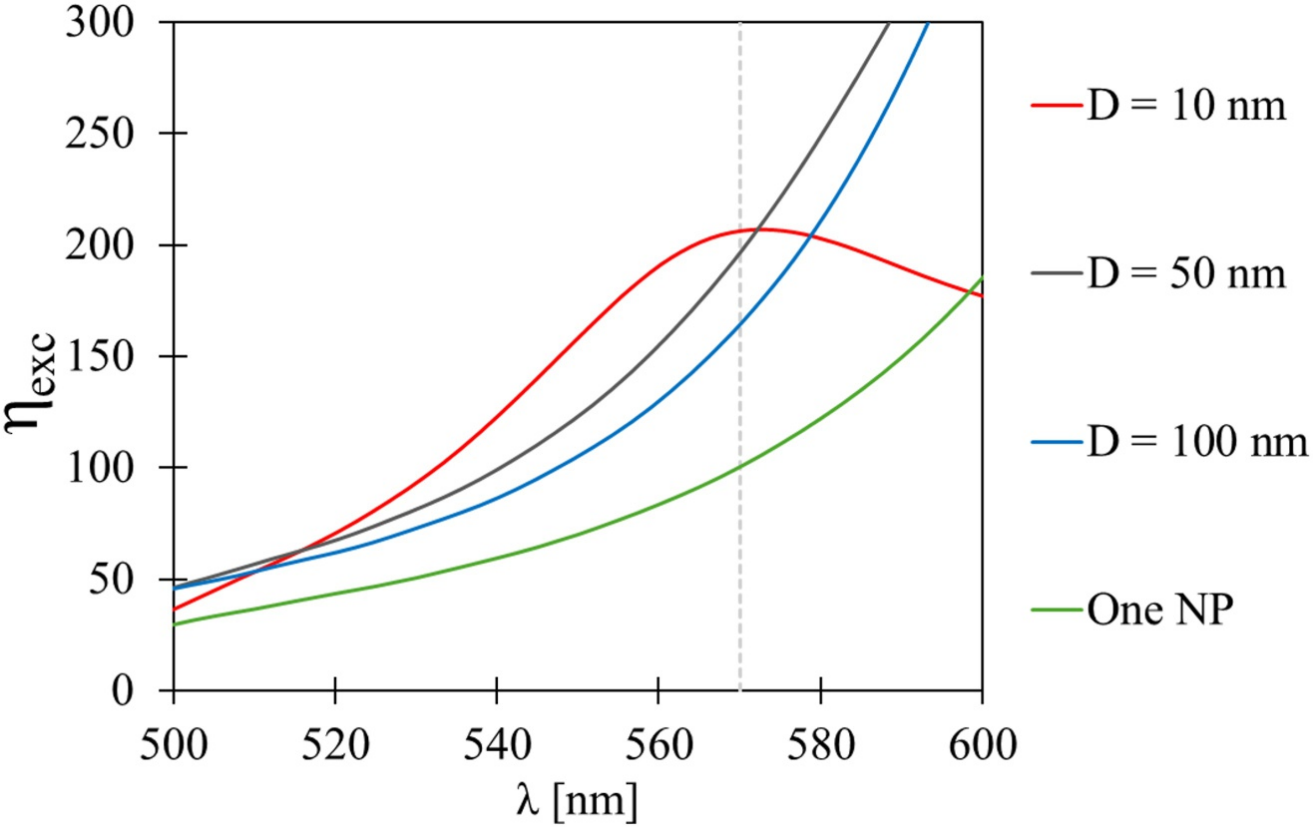
Average excitation enhancement $\langle \eta_{\text{exc}}\rangle$ of the nanostructure, comparing dimer configurations with varying interparticle gaps and the isolated NP. The most relevant cases are highlighted: D=10 nm (red), D=100 nm (blue), and the isolated NP (green). The vertical dashed line indicates the excitation wavelength, $\lambda_{\text{exc}}=570$ nm. The NPs were illuminated by a plane wave linearly polarized along the x‐axis and propagating along the –z‐axis.

At the excitation wavelength (λ=570 nm), the lowest value of the excitation enhancement factor, $\langle \eta_{\text{exc}}\rangle$, corresponds to the single NP configuration. In contrast, the dimer configurations exhibit higher values.

For the case D=10 nm, the maximum of $\langle \eta_{\text{exc}}\rangle$ coincides with the excitation wavelength because of the emergence of a new resonance arising from the near‐field interaction between the NPs, as discussed in the previous section. In this configuration, $\langle \eta_{\text{exc}}\rangle$ reaches a value of 207. As the gap increases, the interaction‐induced resonance gradually weakens, and no distinct spectral feature is observed at the excitation wavelength. Nevertheless, relatively high $\langle \eta_{\text{exc}}\rangle$ values, on the order of 170 and 100, are still obtained for D=100 nm and for the isolated NP, respectively, despite the absence of a resonance at this wavelength. This behavior confirms the beneficial effect of the NP interaction, which induces the formation of an out‐of‐plane electric dipole at the excitation wavelength, resulting in enhanced electric‐field confinement at the QD location.

### 4.4 Emission Enhancement and Directionality

The average emission enhancement $\langle \eta_{\text{em}}\rangle$ in the upward direction for randomly oriented QDs in the spacer layer beneath the NPs was evaluated, considering the spatial distribution of the emitters in the spacer layer as described in the emission enhancement section (see Supporting Information S1: SI‐S4 for further details).

The results are shown in Figure 6, comparing a single NP with dimer configurations with gap sizes D ranging from 10 to 100 nm. At the emission wavelength ($\lambda_{\text{em}}=650$ nm), the highest enhancement was obtained for the single NP, reaching a value of 517.

**FIGURE 6:**
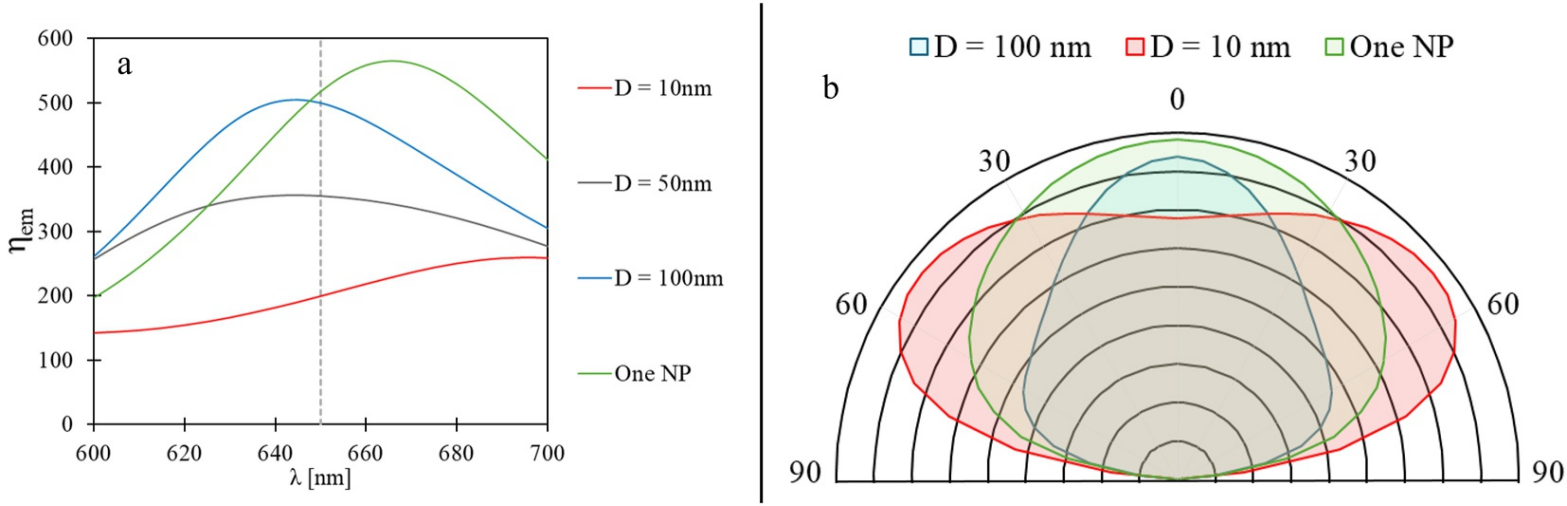
(a) Average emission enhancement $\langle \eta_{\text{em}}\rangle$ spectrum in the upward direction for different gap sizes and the isolated NP. The vertical dashed line indicates the emission wavelength ($\lambda_{\text{em}}=650$ nm). The selected configurations are highlighted: a 10‐nm gap dimer (red), a 100‐nm gap dimer (blue), and a single NP (green). (b) Emission directionality at $\lambda_{\text{em}}=650$ nm for the same configurations.

As D decreases in the dimers, the average emission enhancement progressively decreases, from a value of 500 for the dimer with D=100 nm to 200 for the dimer with D=10 nm. In agreement with the near‐field distributions shown in Figure 4, this reduction can be attributed to the weaker confinement of the electromagnetic energy within the spacer region, resulting in a lower local density of optical states (LDOS).

It has been demonstrated that the nanostructures enhance the radiation emitted by the QDs in the upward (out‐of‐plane) direction. However, it is also important to analyze how the emitted radiation is distributed over the upper hemisphere, that is, within the air region. Given that the objective is to maximize upward emission, highly directive scattering patterns are desired, where the radiation is concentrated within a narrow angular range centered around the surface normal.

The emission directionality pattern at λ=650 nm is shown in Figure 6b. Simulations were performed for representative gap sizes of D=10 and 100 nm, as well as for the isolated NP.

The results indicate that larger gaps can achieve emission enhancements comparable to those of the isolated NP while providing improved directionality. In contrast, for D=10 nm, narrow scattering patterns are not observed due to the strong interaction between the NPs.

To quantitatively evaluate the average directionality enhancement $\langle D_{\text{em}}/D_{\text{em}}^{0}\rangle$, Equation (2) was applied to both the nanoantenna configurations (single NP and dimer) $\langle D_{\text{em}}\rangle$ and the reference case without nanostructures $\langle D_{\text{em}}^{0}\rangle$. The corresponding directionality enhancement values are summarized in Table 1.

**TABLE 1:** Average directionality enhancement for a single NP and dimers with D=10 nm and D=100 nm.

Configuration	$\langle D_{\text{em}}/D_{\text{em}}^{0}\rangle$
Single NP	2.1
D=10 nm	1.8
D=100 nm	2.3

As inferred from the directionality patterns, the highest directionality enhancement is achieved for the dimer with a gap of D=100 nm, being 1.1 and 1.3 times larger than that of the isolated NP and the dimer with D=10 nm, respectively.

### 4.5 Photoluminescence Enhancement

These findings reveal a trade‐off in the dimer configuration: small gaps enhance excitation because of the emergence of a new resonance at the excitation wavelength arising from the interaction between the NPs, whereas larger gaps favor emission enhancement and improved directionality.

Considering that the photoluminescence (PL) enhancement can be expressed as the product of the excitation enhancement and the emission enhancement collected within a given numerical aperture (NA), as defined in Equation (10), which is valid for low intrinsic quantum yield emitters, we estimate the PL enhancement for both the dimer structures and the isolated NP. In this analysis, only the radiation emitted in the upward direction is considered:

(10) $$PL\approx \eta_{\text{exc}}\eta_{\text{em}}$$

Notably, the average PL enhancement is not, in general, equal to the product of the average excitation and emission enhancements in the presence of spatially inhomogeneous coupling between the QDs and the nanoantenna [25]:

(11) $$\langle PL\rangle \neq \langle \eta_{\text{exc}}\rangle \;\langle \eta_{\text{em}}\rangle$$

For this reason, we consider the product of the average excitation and emission enhancements as an approximation to the PL enhancement.

However, a more accurate description requires evaluating the excitation and emission for each dipole orientation and emitter position. For each orientation, the PL is obtained as the product of the excitation and emission, resulting in a spatial map of the PL as a function of the QD position.

Subsequently, the three maps corresponding to the x‐, y‐, and z‐oriented dipoles are averaged to account for randomly oriented emitters. The resulting map is then integrated over all emitter positions and normalized to the corresponding quantity in the absence of the nanoantenna.

In Table 2, we compare the results obtained from this approach 〈PL〉 with those derived from the approximation based on the product of the averaged excitation and emission enhancements $\langle \eta_{\text{exc}}\rangle \langle \eta_{\text{em}}\rangle$ for the isolated NP and the dimer for gaps D=10 and 100 nm.

**TABLE 2:** Comparison of the product of the average excitation enhancement and the average emission enhancement, along with the average PL enhancement, for a single NP and for dimers with D=10nm and D=100nm.

Configuration	$\langle \eta_{\text{exc}}\rangle \langle \eta_{\text{em}}\rangle$	〈PL〉
Single NP	51,700	161,620
D=10 nm	41,400	90,366
D=100 nm	85,000	278,354

The results indicate that the average PL enhancement is between 2 and 4 times larger than the product of the excitation and emission enhancements. Furthermore, it can be observed that using a dimer with a sufficiently large gap between the NPs can outperform the response of an isolated NP, yielding a PL enhancement approximately 1.7 times greater than that of the single NP configuration. However, it is also demonstrated that very small gaps can lead to a reduction in the PL enhancement because of a decrease in the emission enhancement. This behavior can be attributed to the redistribution of the electric field at the emission wavelength. As the gap between the NPs decreases, part of the field becomes increasingly confined within the NP gap region, reducing the field overlap with the spacer layer containing the quantum emitters. As a consequence, the LDOS experienced by the emitters decreases, leading to a reduction in the emission enhancement. Nevertheless, configurations with small NP gaps remain of particular interest due to their enhanced excitation response.

The relative contributions of excitation and emission processes to the overall PL enhancement depend on the properties of the quantum emitter, as can be inferred from Equation (12):

(12) $$PL=\frac{\gamma_{\text{exc}}\left(r\right)}{\gamma_{\text{exc}}^{0}}\frac{D_{\text{em}}\left(r\right)}{D_{\text{em}}^{0}}\frac{QY\left(r\right)}{QY^{0}}$$

where $\gamma_{\text{exc}}$ and $\gamma_{\text{exc}}^{0}$ denote the excitation rates with and without the nanostructure and QY and $QY^{0}$ represent the modified and intrinsic quantum yields, respectively. It should be noted that all the terms in Equation (12) depend on the emitter position r. This spatial dependence arises from the nonuniform electromagnetic environment within the spacer layer, where the emitters are located. This behavior can be observed in Supporting Information S1: SI‐S6.

For efficient emitters, that is, those with high quantum yield operating in the weak excitation regime, the nanoantenna cannot significantly enhance the quantum yield and may even reduce it due to additional nonradiative losses. In this case, the dominant contribution arises from excitation enhancement, which increases the radiated power density.

In contrast, for inefficient emitters, such as the CdSe/ZnS QDs considered in this work, emission enhancement plays a crucial role, as the nanoantenna can effectively increase the radiative decay rate.

From this analysis, it follows that dimer configurations not only outperform isolated NPs but also provide an additional degree of freedom through the gap size, which can be optimized depending on the specific emitter under consideration. Furthermore, the obtained PL enhancement represents an improvement of approximately two orders of magnitude compared with the maximum value reported in Ref. [25], where a theoretical PL enhancement of 1263 was achieved. To place the PL enhancement predicted in this work in the context of previously reported NPoM systems, Table 3 summarizes representative theoretical and experimental results from the literature.

**TABLE 3:** Comparison of the proposed dimer performance with representative nanoparticle‐on‐mirror structures reported in the literature for PL enhancement.

Structure geometry	Approach	PL Enh.	Ref.
Si/Au ring NPoM	Theo./exp.	$∼1.2\times 10^{3}$/$∼6.5\times 10^{2}$	[25]
Ag/Al cube NPoM	Theo./exp.	×480/×330	[36]
Si/Au sphere NPoM	Exp.	$∼7.8\times 10^{2}$	[37]
Ag/Ag cube NPoM	Exp.	$∼3\times 10^{4}$	[38]
Au/Au rod on mirror	Exp.	$∼9\times 10^{2}$	[39]
Au/Au rod on mirror	Theo.	$∼1.2\times 10^{3}$	[8]
Si/Au dimer NPoM	Theo.	Up to $∼2\times 10^{5}$	This work

## 5 Metasurfaces

Beyond the study of nonperiodic nanostructures, metasurfaces based on the same geometries were also investigated (see Supporting Information S1: SI‐S7 for a description of the numerical simulations). Unit cells consisting of single NPs and dimers with interparticle distances of D=10nm and D=100nm were analyzed while systematically varying the lattice periodicity. This approach enables the identification of metasurface parameters that maximize the overall PL enhancement.

When extending the analysis to periodic metasurfaces, the QDs are assumed to remain randomly distributed within the spacer layer, with a statistically uniform average density across the metasurface. Experimentally, QD layers can be deposited using techniques such as spin coating to achieve approximately uniform distributions over large areas and minimize aggregation, although local variations in QD density and localized clustering may occur [25]. Therefore, identical emitter positions in each unit cell are not required. Local variations in the number and positions of QDs between different unit cells are expected to be statistically averaged over a sufficiently large number of emitters and unit cells. However, systematic variations in the QD spatial distribution or density could affect the predicted ensemble‐averaged response, because the excitation enhancement, emission enhancement, and emission directionality depend on the local electromagnetic environment experienced by the emitters.

The reflectivity of the metasurfaces was first evaluated to identify periodic structures exhibiting resonances at the wavelengths of interest, namely, the excitation wavelength (570 nm) and the emission wavelength (650 nm). Figure 7 shows the reflectivity spectra for the periodicities providing the best performance obtained for each configuration: P=450nm for the single NP case, P=500nm for the dimer with D=10nm, and P=650nm for the dimer with D=100nm. Comparing the metasurface response with that of the corresponding isolated nanoantennas reveals a clear influence of the periodic lattice. At the smallest period (P=450nm), the optical response remains similar to that of the isolated NP, exhibiting a comparable resonance wavelength with only a slight narrowing of the spectral linewidth. For the metasurface based on dimers with P=500nm (D=10nm), two resonant features are clearly observed. Although two resonances are also present for the isolated dimer, a direct one‐to‐one correspondence between the resonances of the isolated and periodic systems cannot be generally assumed, because the periodic environment modifies the overall resonant response. In this case, the most evident effect of the periodic arrangement is the considerably narrower linewidth of the resonances. For P=650nm (D=100nm), the resonance around 650nm becomes significantly narrower, whereas an additional sharp resonance appears near 700nm. These spectral modifications arise from collective interactions between the nanoantennas mediated by the periodic lattice. In particular, as the lattice period approaches the wavelength range in which diffractive effects become relevant, the resonances of the individual nanoantennas can couple to the lattice response, giving rise to surface lattice resonances (SLRs), which result in narrower spectral features and the emergence of additional resonances [40].

**FIGURE 7:**
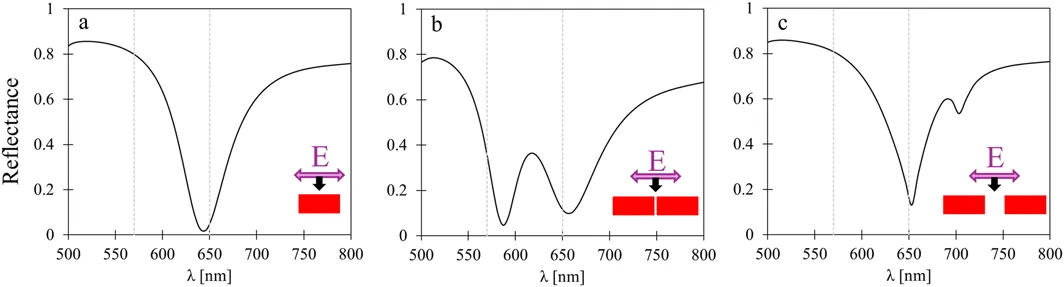
Reflectance spectra of the metasurfaces for (a) an isolated NP with a periodicity of 450 nm, (b) a dimer with D=10 nm and a periodicity of 500 nm, and (c) a dimer with D=100 nm and a periodicity of 650 nm. The vertical dashed lines indicate the excitation (570 nm) and emission (650 nm) wavelengths. The structures were illuminated by a plane wave propagating along the negative z‐direction and linearly polarized along the x‐axis, as shown in the insets.

As in the nonperiodic case, the average excitation enhancement and the average emission enhancement in the upward direction, together with the emission directionality, were evaluated. Figure 8 summarizes these results for the most promising metasurface configurations.

**FIGURE 8:**
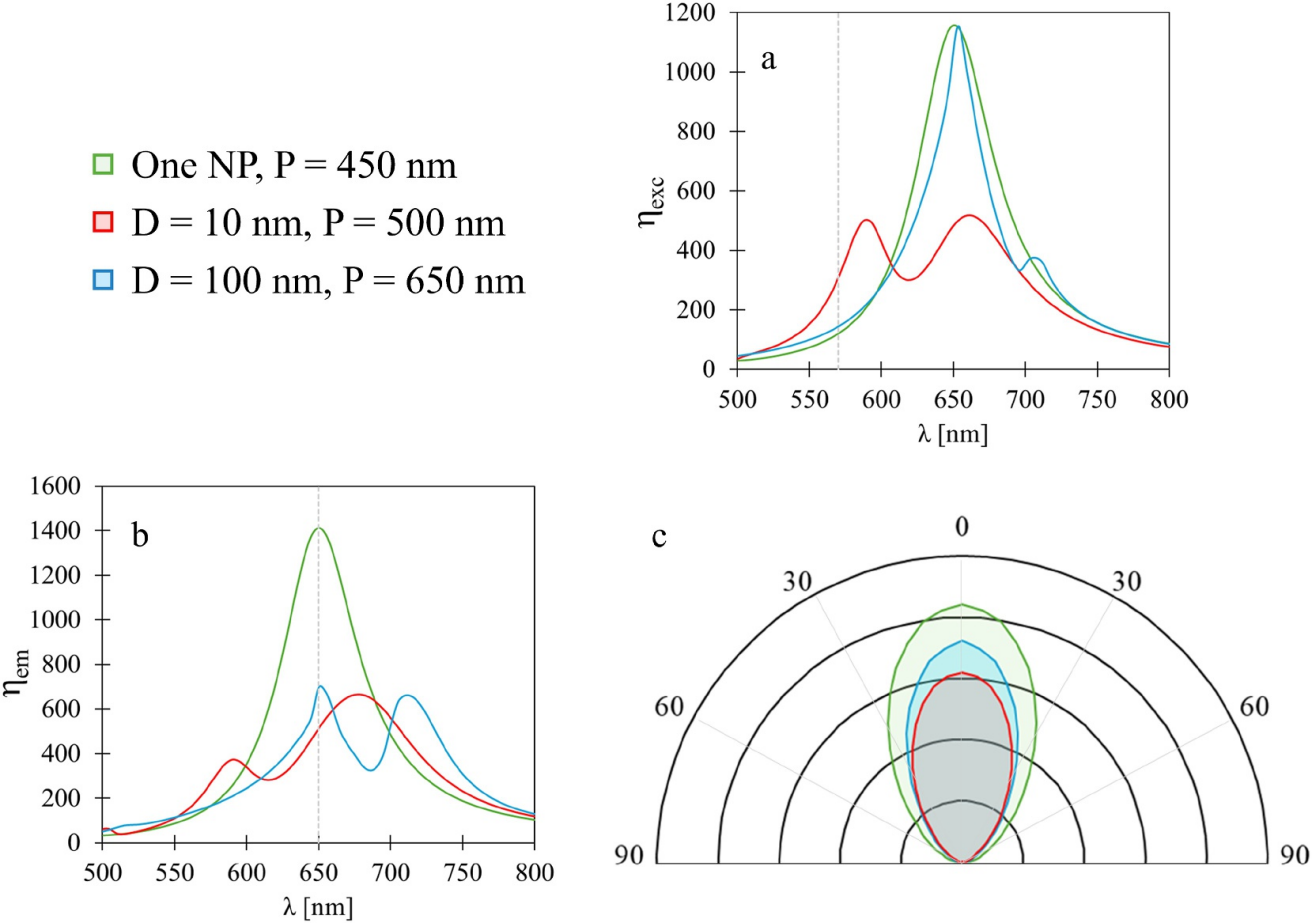
(a) Average excitation enhancement $\langle \eta_{\text{exc}}\rangle$ at λ=570nm, (b) average emission enhancement in the upward direction $\langle \eta_{\text{em}}\rangle$, and (c) emission directionality at λ=650nm for the most promising periodicities. The results correspond to metasurfaces composed of unit cells consisting of a single NP (green), a dimer with a small gap (D=10nm, red), and a dimer with a larger gap (D=100nm, blue).

As observed previously for nonperiodic nanostructures, metasurfaces based on dimers with D=10nm exhibit the highest excitation enhancement, as shown in Figure 8a. This behavior can be attributed to the additional modes arising from the interaction between the two NPs in the dimer, leading to a resonance at the excitation wavelength. For this configuration, the excitation enhancement reaches a value of 325, representing a substantial increase compared with the isolated NP and the D=100nm dimer cases, where the maximum enhancement remains around 120 and 140, respectively.

Regarding the emission enhancement along the normal direction, metasurfaces composed of single NP unit cells provide the highest overall values, exceeding 1400. In contrast, significantly lower enhancements are obtained for metasurfaces based on dimers, with values of 696 and 513 for gaps of D=100nm and D=10nm, respectively.

This behavior contrasts with that observed for the nonperiodic nanostructures, where a similar emission enhancement was obtained for both the isolated NP and the dimer with D=100nm. This effect can be attributed to the fact that the resonance responsible for the enhancement is no longer purely dipolar. Although the response of the isolated nanoparticle and the small‐gap dimer is mainly governed by dipolar modes, higher‐order multipolar contributions emerge in the large‐gap dimer because of the increased periodicity.

The emission directionality was also evaluated. As shown in Figure 8c, all three configurations exhibit directional emission patterns, with the dimers showing a higher degree of directionality than the isolated NP.

Table 4 summarizes the average excitation and emission enhancements, together with the directionality enhancement in the upward direction, as well as the product of the excitation and emission enhancements. This quantity is then compared with the average PL enhancement calculated as described in the previous section. The highest PL is obtained for the isolated NP, followed by the dimer with D=10nm.

**TABLE 4:** Comparison of the average excitation enhancement, the average upward emission enhancement, the average directionality enhancement in the upward direction, the product of the average excitation and emission enhancements, and the average PL enhancement for the most efficient metasurfaces based on a single NP and dimers with D=10nm and D=100nm.

Configuration	$\langle \eta_{\text{exc}}\rangle$	$\langle \eta_{\text{em}}\rangle$	$\langle D_{\text{em}}/D_{\text{em}}^{0}\rangle$	$\langle \eta_{\text{exc}}\rangle \langle \eta_{\text{em}}\rangle$	〈PL〉
Single NP—P=450 nm	119	1405	2.635	167,195	482,988
D=10 nm—P=500 nm	325	513	2.7	166,725	381,105
D=100 nm—P=650 nm	144	696	2.73	100,224	224,939

For metasurfaces, a similar behavior is observed as in nonperiodic nanostructures. Controlling the gap between the NPs in a dimer allows tuning the trade‐off between excitation and emission enhancements, enabling the design of configurations optimized for the specific emitter under consideration.

Furthermore, the values obtained for the metasurfaces are larger than those of the corresponding nonperiodic configurations, highlighting the improved performance of metasurfaces in terms of PL enhancement.

## 6 Conclusions

In this work, we investigate hybrid metal‐dielectric nanoantenna configurations consisting of silicon nanoparticle dimers placed on a metallic substrate to enhance the photoluminescence of quantum dots located in the spacer region between the nanoparticles and the substrate. By combining semianalytical modeling based on image theory with numerical simulations, we identify the key physical mechanisms governing the system's response.

Our results demonstrate that the interaction between nanoparticles in the dimer leads to the emergence of additional modes, including out‐of‐plane electric dipole contributions, which play a crucial role in enhancing electromagnetic field confinement within the spacer region. This effect significantly increases the excitation efficiency, particularly for small interparticle gaps. In contrast, larger gaps favor emission enhancement and improved directionality, revealing a clear trade‐off between excitation and emission processes.

By evaluating the photoluminescence enhancement, we show that dimer configurations outperform isolated nanoparticles. Furthermore, the dimer configuration introduces an additional degree of freedom compared with isolated nanoparticles, namely, the interparticle gap. In particular, either excitation or emission enhancement can be selectively optimized depending on the gap size, which may be advantageous depending on the type of quantum emitter under consideration. For emitters with high quantum yield, enhancing excitation is more beneficial, whereas for low quantum yield emitters, emission enhancement plays a more significant role.

Furthermore, the extension of these concepts to periodic metasurfaces demonstrates that enhanced light–matter interaction can be preserved at the collective level. Metasurface configurations enable simultaneous enhancement of excitation and emission while preserving directional radiation, making them attractive for large‐area implementations. In addition, the photoluminescence obtained for the metasurfaces surpasses that of nonperiodic nanostructures.

Overall, these findings provide practical design guidelines for engineering high‐performance photoluminescent nanostructures, with potential applications in single‐photon sources for quantum technologies, fluorescence‐based sensing, and next‐generation nanophotonic light sources.

## Author Contributions

**Rafael Ramos Una:** software, methodology, writing – original draft, data curation, formal analysis, visualization, investigation. **Braulio Garcia‐Camara:** writing – review and editing, formal analysis, supervision. **Angela I. Barreda:** supervision, resources, data curation, software, formal analysis, writing – review and editing, writing – original draft, funding acquisition, conceptualization, methodology.

## Supporting information


Supporting Information S1

## Data Availability

The data that support the findings of this study are available from the corresponding author upon reasonable request.
