# Supplementary material for: Dielectric Nanoparticle Dimers on a Mirror for Enhanced Photoluminescence of Quantum Dots
Source: Nanophotonics. 2026 Oct 14;15(20):e70319. doi: 10.1002/nap2.70319 (PMC13662634; doi:10.1002/nap2.70319)
Supplement: Supplementary file 1 [file NAP2-15-e70319-s001.pdf]

Dielectric Nanoparticle Dimers on a Mirror for Enhanced Photoluminescence of Quantum Dots

Rafael Ramos Uña
Braulio García-Cámara
Ángela I. Barreda

Rafael Ramos Uña, Braulio García-Cámara, Ángela I. Barreda
Address
Department of Electronic Engineering,University Carlos III of Madrid, Avda. de la Universidad, 30, 28911 Leganés, Spain
Email Address: raramosu@ing.uc3m.es, abarreda@ing.uc3m.es

1 SI-S1 Optical constants

As mentioned in the main text, the optical constants used for silicon and aluminum were taken from the Handbook of Optical Constants of Solids (Palik) [1]. Figure SI-1 shows the original data together with the fitted curves employed in the simulations.

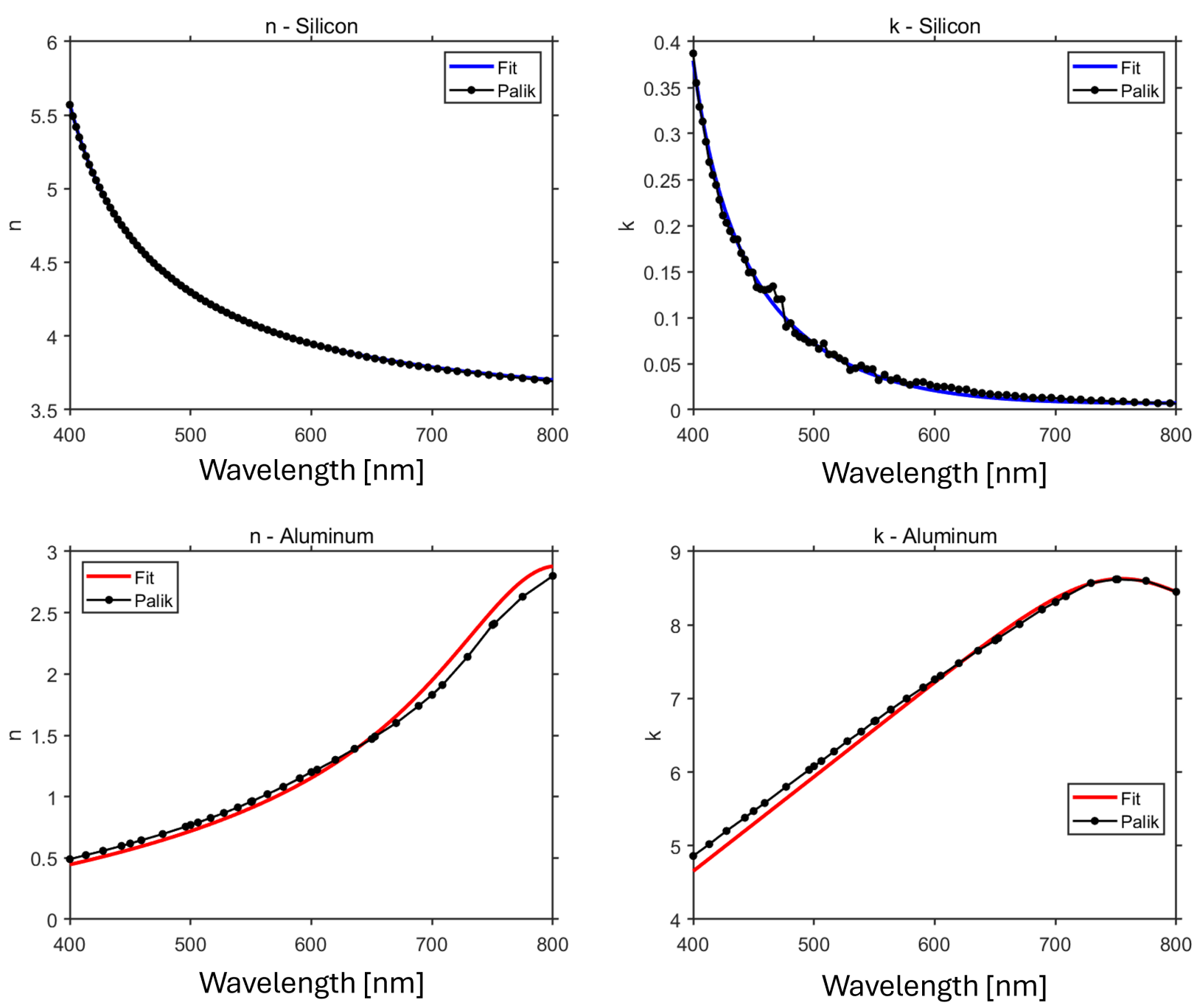

Figure 1: Optical constants for silicon and aluminum alongside with the fit employed in the simulations.

2 SI-S2 Analytical electric and magnetic dipole interaction model for dielectric spherical nanoparticle dimers

Dielectric NPs are known to exhibit distinct electric and magnetic resonant modes driven by displacement currents [2, 3]. Moreover, due to the interaction between the NPs forming a dimer, new electric and magnetic dipolar, or even higher-order multipolar, modes can emerge. Within the dipolar approximation, it has been previously shown that, for sufficiently small gap sizes D, out-of-plane electric or magnetic dipole moments can be induced depending on the polarization of the incident radiation [4].

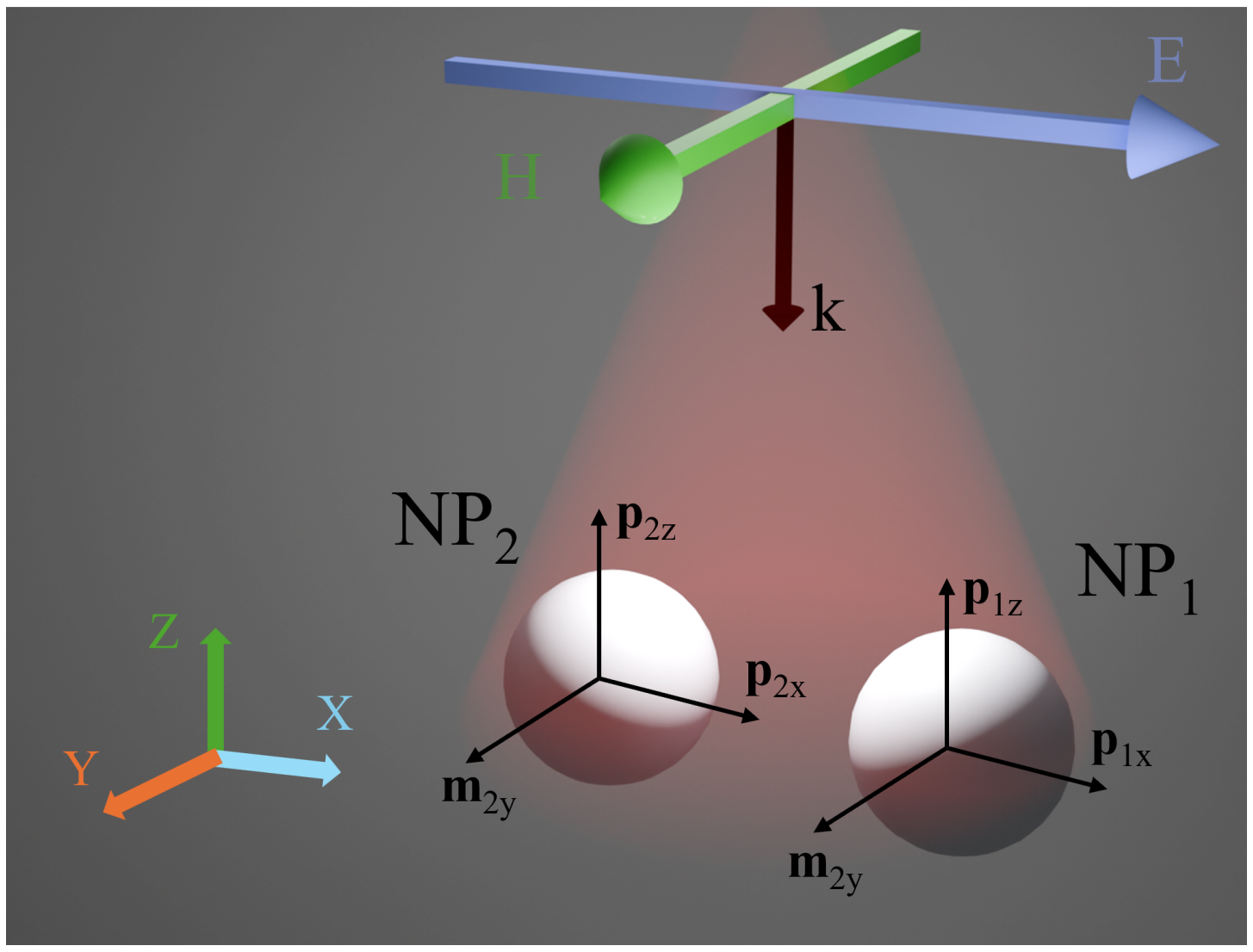

Figure 2: Schematic representation of the dipolar system employed to model the electromagnetic response of a silicon NP dimer. The structure is illuminated by a plane wave propagating along the negative z-axis and linearly polarized along the x-axis (p-polarization). The silicon NPs are represented by an equivalent distribution of point dipoles p_{1x}, p_{2x}, m_{1y}, m_{2y}, p_{1z}, p_{2z}.

To analytically describe this effect, the NPs are assumed to be spherical, so that each of them can be modeled as co-located electric and magnetic dipoles, mutually orthogonal and perpendicular to the propagation direction of the incident field, and positioned at the center of mass of each NP (see Fig. SI-2). The interaction between the particles is then treated using the Green's function formalism [5].

In this section, we analyze the interaction effects between two silicon nanospheres of radius $R = 70$ nm, separated by different gap distances D and located at $\mathbf{r}_1 = (+D/2 + R, 0, 0)$ and $\mathbf{r}_2 = (-D/2 - R, 0, 0)$. The system is excited by a plane wave propagating along the negative z-direction and linearly polarized along the x-axis, which coincides with the dimer axis (p-polarization). The induced dipoles arise from both the incident field and the field scattered by the neighboring NP.

Following the analytical dipole–dipole interaction model described in [4, 6], we calculate the electric and magnetic dipole moments induced in each NP. These induced dipole moments are then used to compute the extinction cross-section of the system.

For p-polarization, the induced dipole moments satisfy the following self-consistent relations [6]:

$$p_{1x} = \varepsilon_0 \varepsilon_h \alpha_e E_0 + \alpha_e k^2 g_{xx}\, p_{2x}, \tag{1}$$

$$p_{2x} = \varepsilon_0 \varepsilon_h \alpha_e E_0 + \alpha_e k^2 g_{xx}\, p_{1x}, \tag{2}$$

$$p_{1z} = -\alpha_e k^2 g_{yy}\, p_{2z} + i\varepsilon_0 \varepsilon_h \alpha_e Z k^2 g_{zy}\, m_{2y}, \tag{3}$$

$$p_{2z} = -\alpha_e k^2 g_{yy}\, p_{1z} - i\varepsilon_0 \varepsilon_h \alpha_e Z k^2 g_{zy}\, m_{1y}, \tag{4}$$

$$m_{1y} = \alpha_m \left(-\frac{E_0}{Z} + \frac{ik^2}{Z\varepsilon_0 \varepsilon_h} g_{zy}\, p_{2z} - k^2 g_{yy}\, m_{2y} \right), \tag{5}$$

$$m_{2y} = \alpha_m \left(-\frac{E_0}{Z} - \frac{ik^2}{Z\varepsilon_0 \varepsilon_h} g_{zy}\, p_{1z} - k^2 g_{yy}\, m_{1y} \right). \tag{6}$$

being

$$g_{yy} = -\left(1 + \frac{i}{kD} - \frac{1}{k^2D^2}\right) g(D) \tag{7}$$

$$g_{zy} = -\left(i - \frac{1}{kD}\right) g(D) \tag{8}$$

$$g_{xx} = \left(-\frac{2i}{kD} + \frac{2}{k^2D^2}\right) g(D) \tag{9}$$

where $g(D) = \frac{e^{ikD}}{4\pi D}$ is the scalar Green's function
In this illumination geometry, the primary moments are p_x and m_y. The out-of-plane electric dipoles p_{1z} and p_{2z} are not directly driven by the incident field and arise from the mutual interaction terms proportional to g_{zy}, which couple m_y into p_z [6].
By introducing the dressed polarizabilities, which are related to the electric (α_e) and magnetic (α_m) dipole polarizabilities, as follows:

$$\tilde{\alpha}_{ex} \equiv \frac{\alpha_e}{1-\alpha_e k^2 g_{xx}}, \quad \tilde{\alpha}_{ez} \equiv \frac{\alpha_e}{1-\alpha_e k^2 g_{yy}}, \quad \tilde{\alpha}_{my} \equiv \frac{\alpha_m}{1+\alpha_m k^2 g_{yy}} \tag{10}$$

The expressions from Eqs. 1 to 6 can then be derived, as described in [6], and collectively written as:

$$p_{1x} = p_{2x} = \varepsilon_0 \varepsilon_h \tilde{\alpha}_{ex} E_0 \tag{11}$$

$$p_{1z} = -p_{2z} = -i\epsilon_0\varepsilon_h E_0 \frac{\tilde{\alpha}_{my}\tilde{\alpha}_{ez}k^2 g_{zy}}{1-\tilde{\alpha}_{my}\tilde{\alpha}_{ez}k^4 g_{zy}^2} \equiv -i\epsilon_0\varepsilon_h E_0 \tilde{\alpha}_{e-m} \tag{12}$$

$$m_{1y} = m_{2y} = -\frac{\tilde{\alpha}_{\mathrm{my}}}{Z} E_0 - \left(\tilde{\alpha}_{e-m} k^2 g_{zy}\right) \frac{\tilde{\alpha}_{\mathrm{my}}}{Z} E_0 \tag{13}$$

The extinction cross-section is given by:

$$\sigma_{\mathrm{ext}} = 2k\,\mathrm{Im}\left\{\tilde{\alpha}_{ex} + \tilde{\alpha}_{my} + \left(\tilde{\alpha}_{(e-m)} k^2 g_{zy}\right)\tilde{\alpha}_{my}\right\}. \tag{14}$$

The extinction cross-section can be expressed as the sum of the scattering (σ_{sca}) and absorption (σ_{abs}) cross-sections:

$$\sigma_{\mathrm{ext}} = \sigma_{\mathrm{sca}} + \sigma_{\mathrm{abs}} \tag{15}$$

Figure SI-3 shows the extinction efficiency spectra of a dimer composed of spheres with radius $R = 70$ nm, chosen to match the volume of the nanocylinders used in this work. The spectra are calculated from Eq. 14 by normalizing the extinction cross-section to the geometric cross-sectional area of the NPs (σ_{geo}). The electric and magnetic dipolar contributions are also shown. Results are presented for different nanoparticle separations D under p-polarized illumination. For comparison, the spectra obtained from FDTD simulations are also included.
As shown in Fig. SI-3, the induced out-of-plane electric dipole arising from the interaction between the NPs is particularly strong at small separations and gradually decreases as the distance between them increases (see red box in Fig. SI-3a).

3 SI-S3 Excitation enhancement

To evaluate this enhancement, a power monitor was placed at the center of the spacer layer, covering an area equivalent to the effective area defined by the nanoantenna, without considering the gap between

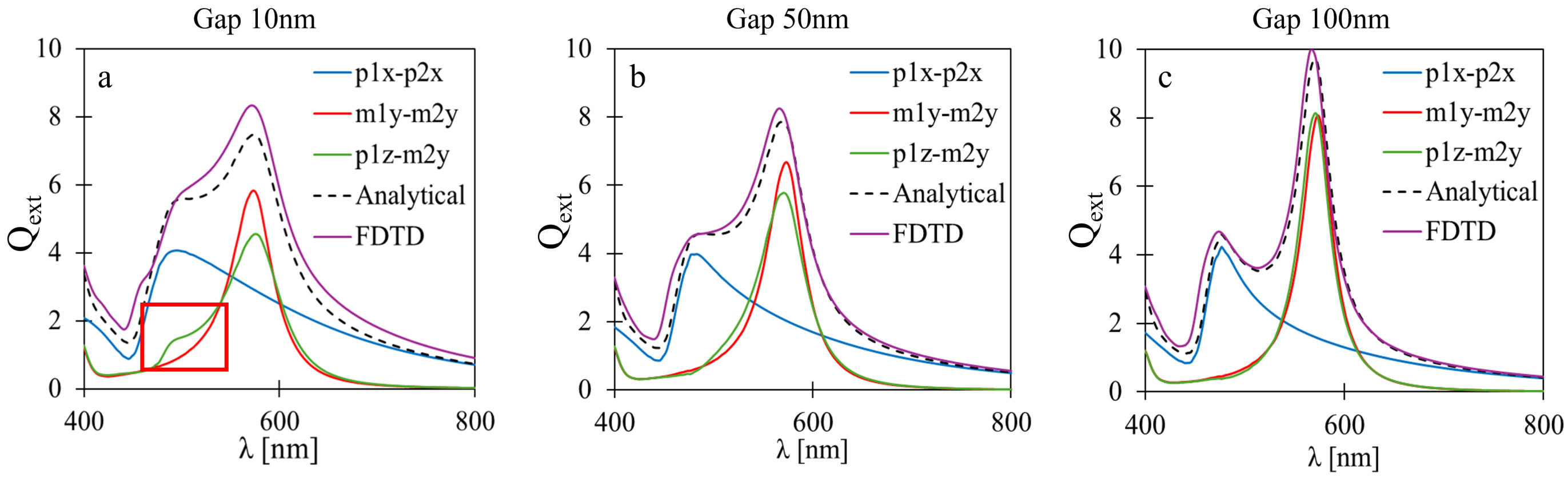

Figure 3: Extinction efficiency spectra calculated using the Green's function formalism and compared with FDTD results for a dimer composed of silicon spheres with a radius of 70 nm. The NPs are illuminated by a plane wave propagating along the negative z-direction and linearly polarized along the x-axis. The interaction between the NPs is analyzed for separations of (a) $D = 10$ nm, (b) 50 nm, and (c) 100 nm. The contribution arising from the interaction between electric dipoles is shown by the blue line, the contribution associated with the interaction between magnetic dipoles by the red line, and the contribution resulting from the interaction between electric and magnetic dipoles by the green line. The dashed black line represents the analytically obtained extinction efficiency, while the purple line corresponds to the extinction efficiency calculated using the FDTD method. The red box in (a) highlights the appearance of the out-of-plane electric dipole induced by the interaction between the NPs.

the NPs in the case of the dimers. The magnitude of the electric field was recorded, averaged over the surface defined by the power monitors, squared, and subsequently normalized to the corresponding value obtained in the absence of the nanoantenna.

4 SI-S4 Emission enhancement and directionality

This approach employs two separate plane-wave sources with orthogonal linear polarizations incident normally onto the nanoantenna. The electric field is recorded in the middle plane of the spacer region beneath the nanoantenna, where the QDs may be located. This corresponds to the same area used in the excitation enhancement simulations, but here sampled using point monitors distributed across the region. In this way, the emission of randomly localized and oriented emitters is emulated. Each nanoparticle was assigned 400 point monitors to ensure accurate spatial sampling.

5 SI-S5 Scattering and absorption efficiencies

The numerical scattering efficiency of the nanoantennas placed on a metallic substrate and separated from it by a dielectric spacer was calculated using the FDTD method. A TFSF source was employed to generate linearly polarized broadband light under normal incidence. The normalized scattering cross-section was defined as $Q_{\text{sca}} = \sigma_{\text{sca}}/\sigma_{\text{geo}}$, where σ_{sca} was obtained by integrating the Poynting vector of the scattered field over power monitors located outside the source region and normalizing the scattered power to the incident intensity. Only the power scattered into the air region was calculated by integrating the scattered field over the corresponding upper boundaries. Perfectly matched layers (PMLs) were used as absorbing boundaries. The same procedure was followed to calculate the absorption efficiency, with the only difference that, for determining the absorption cross-section in *Lumerical*, the power monitor was placed inside the TFSF region.

6 SI-S6: Spatial dependence of the optical response

The optical response depends on the spatial position $\mathbf{r}$ of the QD within the spacer layer due to the nonuniform electromagnetic environment in the region where the emitters are located. Consequently, QDs located at different positions can exhibit different excitation and emission enhancements, as well as different emission directionality, ultimately resulting in a position-dependent PL enhancement. Figure SI-4 illustrates this spatial dependence by mapping the excitation, emission, and resulting PL enhancements across the spacer plane below one of the nanoparticles for three representative configurations: an isolated NP (a-c), a dimer with a 10 nm gap (d-f), and a dimer with a 100 nm gap (g-i).

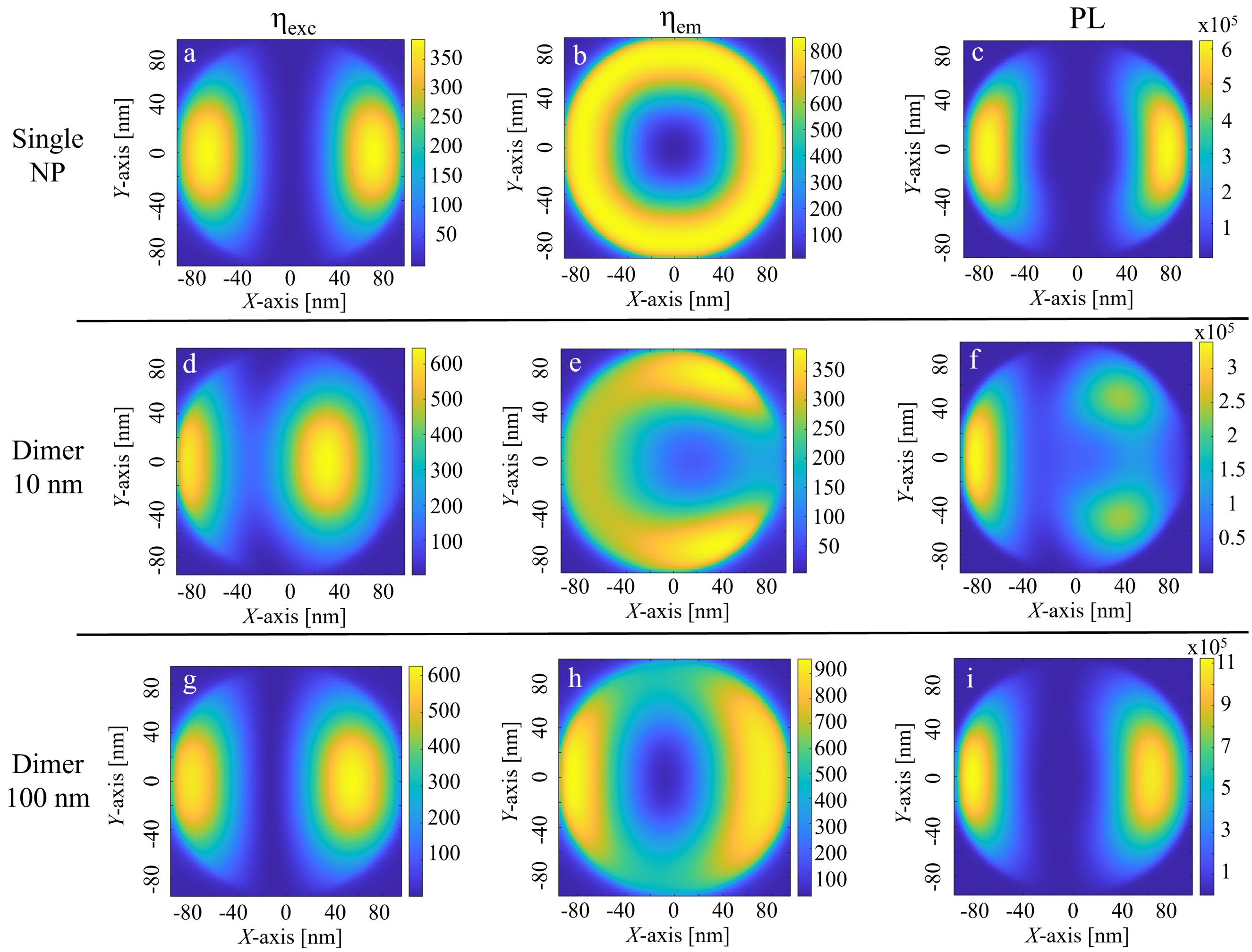

Figure 4: Spatial distribution of the excitation enhancement, $\eta_{\mathrm{exc}}(\mathbf{r})$, at $\lambda_{\mathrm{exc}} = 570$ nm (left column); emission enhancement, $\eta_{\mathrm{em}}(\mathbf{r})$, at $\lambda_{\mathrm{em}} = 650$ nm along the direction normal to the substrate (middle column); and the resulting PL enhancement (right column), evaluated across the spacer plane below one of the nanoparticles. Results are shown for (a–c) an isolated NP, (d–f) a dimer with a 10 nm gap, and (g–i) a dimer with a 100 nm gap. The excitation and emission enhancements are averaged over the x-, y-, and z-oriented dipole contributions, while the PL enhancement is obtained by multiplying the corresponding excitation and emission contributions for each orientation before averaging over the three orientations.

7 SI-S7 Metasurface numerical simulations

The metasurfaces were analyzed by simulating a single unit cell with periodic boundary conditions applied along the x and y directions. PMLs were used along the out-of-plane (z) direction to suppress spu-

rious reflections. The structure was illuminated from the top by a linearly polarized plane wave at normal incidence.

References

[1] Edward D Palik. Handbook of optical constants of solids. In *Handbook of Optical Constants of Solids*. Academic Press, 1998.

[2] Mikhail V. Rybin and Yuri Kivshar. Metaphotonics with subwavelength dielectric resonators. *npj Nanophotonics*, 1(1):43, November 2024.

[3] Jiahui Xu, Yue Wu, Pinzheng Zhang, Yiming Wu, Renaud A. L. Vallée, Suli Wu, and Xiaogang Liu. Resonant Scattering Manipulation of Dielectric Nanoparticles. *Advanced Optical Materials*, 9(15):2100112, August 2021.

[4] Ángela I. Barreda, Hassan Saleh, Amelie Litman, Francisco González, Jean-Michel Geffrin, and Fernando Moreno. Electromagnetic polarization-controlled perfect switching effect with high-refractive-index dimers and the beam-splitter configuration. *Nature Communications*, 8(1):13910, January 2017.

[5] C. Yeh. Dyadic Green's functions in electromagnetic theory. *IEEE Antennas and Propagation Group Newsletter*, 14(3):7–7, September 1972.

[6] Pablo Albella, M. Ameen Poyli, Mikolaj K. Schmidt, Stefan A. Maier, Fernando Moreno, Juan José Sáenz, and Javier Aizpurua. Low-Loss Electric and Magnetic Field-Enhanced Spectroscopy with Subwavelength Silicon Dimers. *The Journal of Physical Chemistry C*, 117(26):13573–13584, July 2013.
